# Neurological outcomes after extracorporeal cardiopulmonary resuscitation in patients with out-of-hospital cardiac arrest: a retrospective observational study in a rural tertiary care center

**DOI:** 10.1186/2052-0492-2-33

**Published:** 2014-06-02

**Authors:** Katsunori Mochizuki, Hiroshi Imamura, Tomomi Iwashita, Kazufumi Okamoto

**Affiliations:** Department of Emergency and Critical Care Medicine, Shinshu University School of Medicine, 3-1-1 Asahi, Matsumoto, 390-8621 Japan

**Keywords:** Out-of-hospital cardiac arrest, Cardiopulmonary resuscitation, Extracorporeal cardiopulmonary resuscitation, Cardiac arrest bypass

## Abstract

**Background:**

In a rural region with few medical resources, we have promoted the strategy that if an out-of-hospital cardiac arrest (OHCA) patient is likely reversible, he or she should be transported directly from the scene of cardiac arrest to the only tertiary care center where extracorporeal cardiopulmonary resuscitation (ECPR) is readily available. We investigated 1-month survival and neurological outcomes after ECPR in OHCA patients at this center.

**Methods:**

We implemented a retrospective review of OHCA patients of heterogeneous origin in whom ECPR was performed. Demographic characteristics, cardiopulmonary resuscitation, ECPR details, and neurological outcomes were evaluated. Cerebral performance categories were used to assign each patient to favorable or unfavorable outcome groups.

**Results:**

Fifty OHCA patients underwent ECPR. Presumed causes of OHCA were cardiac etiology in 32 patients, accidental hypothermia in 7 patients, and other causes in 11 patients. Overall, 13 patients (26%) survived and 10 patients (20%) had favorable outcomes. Of the 32 patients with OHCA of cardiac origin, 5 patients (16%) had favorable outcomes. Of the seven patients with OHCA of hypothermic origin, five patients (71%) had favorable outcomes. No clinically reliable predictors to identify ECPR candidates were found. However, all nine OHCA patients over 70 years of age had unfavorable outcomes (*P* = 0.224). In addition, all seven patients who satisfied the basic life support termination-of-resuscitation rule had unfavorable outcomes (*P* = 0.319).

**Conclusions:**

ECPR can be a useful means to rescue OHCA patients who are unresponsive to conventional cardiopulmonary resuscitation in a rural tertiary care center, in a manner similar to that observed in the urban regions.

## Background

Out-of-hospital cardiac arrest (OHCA) is a socio-economic issue. In Japan, approximately 127,000 OHCAs occur per year [1], and the number of OHCA events is increasing annually. The 1-month survival rate for OHCA patients is approximately 5% [2, 3], and the rate of neurologically favorable 1-month survival is only approximately 2% [2, 3]. Among survivors, over 50% have severe cerebral disability [2, 3]. Every year approximately 4,000 patients survive with severe cerebral disability [2, 3]. The goal of cardiopulmonary resuscitation (CPR) for patients suffering from cardiac arrest (CA) is to achieve neurologically intact survival.

Extracorporeal CPR (ECPR) is a technique to circulate blood outside the body with extracorporeal oxygenation and support the body's circulation in the absence of an adequately functioning cardiac pump. Encouraging results of ECPR for CA of cardiac origin have been reported for in-hospital cardiac arrest (IHCA) in adults and children [4, 5]. The rates of neurologically favorable IHCA survival have been reported as 20% in adults [4] and 38% in children [5]. The favorable results obtained in IHCA patients cannot be directly extrapolated to OHCA patients because of longer transport times and possible delay in initiating ECPR. The rates of neurologically favorable survival after OHCA of cardiac origin have been reported as 12% and 15% in studies of urban regions [6, 7].

Our hospital is the only university tertiary care center where ECPR and comprehensive post-CA care are readily available in the rural region. To minimize the delay in the initiation of ECPR, we have been promoting a proposal, the CA bypass campaign, that emergency medical service (EMS) personnel should bypass the nearest general hospital and directly transport an OHCA patient from the scene to our center when the OHCA patient is likely reversible. Implementation of regional systems of care for those with acute myocardial infarction and trauma has been reported to improve outcomes [8, 9]. Accordingly, we hypothesized that the implementation of ECPR in a rural region might lead to favorable neurological outcomes, similar to that observed in urban region.

In this study, we investigated 1-month survival and neurological outcomes after ECPR in OHCA patients unresponsive to conventional CPR in this region, where the CA bypass campaign has been gradually implemented.

## Methods

We conducted a retrospective review of the Advanced Emergency and Critical Care Center database of Shinshu University Hospital from April 2004 to March 2013 and selected all patients, 10 years of age or older and supported with ECPR for OHCA, who were transported after failed conventional CPR including defibrillation. Review of patient medical records was approved by the Ethics Review Board of Shinshu University School of Medicine, and the need for informed consent was waived. For the purpose of this study, we defined ECPR as extracorporeal membrane oxygenation (ECMO) instituted during chest compressions in the emergency room. Patients who required ECMO support in the course of post-CA care in the intensive care unit were excluded.

### EMS in the Matsumoto region

Emergency medical services (EMS) in the Matsumoto region covers 1,869 km^2^ including three small cities and five villages with a population of approximately 430,000. The emergency lifesaving technician (ELT) has been permitted to use semiautomated external defibrillators for OHCA patients since May 2003—i.e., before the study period. Certified ELTs have been permitted to perform tracheal intubation for OHCA patients since November 2004 and to administer intravenous epinephrine since April 2006. The CA bypass strategy proposed by our department for use by the EMS has been gradually accepted by EMS personnel and implemented over the past 10 years.

### Immediate on-admission ECPR

Patients with OHCA who did not respond to conventional CPR including defibrillation and other lifesaving techniques by EMS personnel were eligible for ECPR. The decision to deploy ECPR was made by an attending physician assigned to the emergency room, based on preliminary information from EMS personnel prior to ambulance arrival at the center. It consisted mainly of age, electrocardiogram, presumed cause of CA, witnessed or unwitnessed CA, presence or absence of bystander CPR, and activities of daily living. The attending physician acted as the event manager responsible for overseeing all aspects of CPR and ensuring resource availability.

ECPR was actively instituted when patients satisfied one of the following conditions:

1. CA caused by accidental hypothermia
2. Witnessed CA followed by bystander CPR and presence of ventricular fibrillation (VF)/tachycardia (VT) on arrival at the emergency room in patients <70 years old with potentially correctable conditions
3. CA in young patients

Exclusion criteria for the initiation of ECPR included the following:

1. Severe trauma
2. Uncontrollable hemorrhage
3. Terminal malignancy
4. Irreversible brain damage
5. Severe activity-of-daily-living disability
6. Signed ‘do not attempt resuscitation’ order

However, it was generally difficult to obtain precise information before ECPR in most patients. To minimize the delay in the initiation of ECPR, the final decision to implement ECPR was left to each attending physician's judgment based on whether or not the OHCA patient was likely reversible.

The main component of the ECPR system consisted of a heparin-bonded membrane oxygenator and circuit (CAPIOX, Terumo Corp, Tokyo, Japan), percutaneous cannulae, and a centrifugal pump (SP45 or SP101, Terumo Corp, Tokyo, Japan). The ECPR system with necessary surgical instruments was always available in the center. In general, the membrane oxygenator and circuit were assembled and primed with saline containing unfractionated heparin ahead of OHCA patients' arrival at the center. The ECMO circuit was connected to a heat exchanger to induce hypothermia.

ECMO was instituted by the ECPR team, which was composed of emergency physicians, intensivists, cardiologists, clinical engineers, emergency center residents, and nursing staff. The attending physician assigned each team member to 1 of 4 groups: a CPR group, an ECMO cannulation group, a centrifugal pump group, and a circulating nurse group. Conventional CPR was continued until the start of ECMO. A percutaneous cannulation was established in the femoral vein (cannula size, mainly 21 F) and artery (cannula size, mainly 15 F). All ECPR patients were supported with veno-arterial ECMO. Femoral cut-down procedures were performed if required. To avoid lower limb ischemia, an anterograde reperfusion catheter for distal limb perfusion was inserted as necessary [10]. After initiation of ECPR, ECMO flow was increased to achieve a target rate of 60 mL/kg/min. Sweep oxygen flow for the membrane oxygenator was initiated at the same flow rate as the blood flow and then adjusted to maintain a PaCO_2_ of 35–40 mmHg.

### Post-CA care

After the start of ECMO, we immediately implemented post-CA treatments consisting of hemodynamic and gas-exchange optimization, therapeutic hypothermia if patients lacked meaningful response to verbal commands and if not hypotensive, emergency percutaneous coronary intervention (PCI) if CA was likely caused by acute myocardial infarction, and glycemic control. An intra-aortic balloon pump (IABP) was also used, if required, for hemodynamic stability. Transthoracic echocardiography was frequently performed by cardiologists to monitor left ventricular function and potential intraventricular thrombus formation. Therapeutic hypothermia was maintained at 33°C to 34°C for 24 h, and rewarming was then conducted gradually for 2 days. During therapeutic hypothermia, midazolam, fentanyl, and vecuronium were used to facilitate therapeutic hypothermia and control shivering. Anticoagulation was provided using unfractionated heparin infusion to maintain an activated clotting time of approximately 200 s. Serial neurological examination was also performed in addition to electroencephalogram and auditory brain stem response. ECMO was discontinued when the patient was hemodynamically stable and adequately oxygenated under ECMO flow of 1 L/min. Cessation of ECMO was considered if severe neurological impairment persisted for more than 7 days without signs of recovery.

### Data collection

We collected data on patient demographics and prehospital variables from the registry of the Matsumoto region EMS and data on in-hospital variables from the medical registry of our center. The data collected were age, sex, past history, witnessed or unwitnessed CA, presence or absence of bystander CPR, presumed cause of CA, and initial cardiac rhythm on the scene and on arrival at the center, along with body temperature, pupil diameter, and laboratory results on arrival at the center.

We further recorded presumed circulatory arrest time defined as the interval from CA to CPR initiation, CPR duration defined as the interval from start to finish of chest compression, door to ECPR initiation time defined as the interval from arrival at the center to ECPR initiation, and ECMO duration. ECPR-related complications, return of spontaneous circulation (ROSC), and survival or death were also collected. ROSC was defined as the obvious presence of intra-arterial waveform by spontaneous circulation.

As reliable criteria for termination of resuscitation (TOR) efforts in adult OHCA cases at the scene, a basic life support TOR rule (BLS-TOR rule) and an advanced life support (ALS) TOR rule (ALS-TOR rule) are recommended [11, 12]. The BLS-TOR rule must satisfy all three of the following criteria: (1) event not witnessed by EMS personnel, (2) no automated external defibrillator used or manual shock applied in out-of-hospital setting, and (3) no ROSC in out-of-hospital setting. The ALS-TOR rule must satisfy all three of the following criteria: (1) the BLS-TOR rule, (2) CA not witnessed by a bystander, and (3) no bystander CPR. The BLS and ALS rules were also checked in each patient in this study.

### Outcome measures

The primary outcome of this study was the favorable neurological status at 1 month after CA. The functional status of survivors at 1 month after CA was followed up according to the Glasgow-Pittsburgh cerebral performance categories (CPC) score [13]. Favorable outcome was defined as CPC score of 1 or 2. Unfavorable outcome was defined as CPC scores of 3, 4, or 5.

### Statistical analysis

Demographic, prehospital, and in-hospital variables were compared between the favorable and unfavorable outcome groups. Categorical variables were compared by the Chi-square test and Fisher's exact tests (when case numbers were small). Continuous variables were compared between groups using Student's *t* test. Binary logistic regression analysis was performed to determine the independent predictive values for the outcome. Descriptive statistics were reported as means ± standard deviation for continuous variables and as frequencies and percentages for categorical variables. Analyses of data were performed using statistical software (IBM SPSS Statistics version 22, SPSS. Japan Inc., Tokyo, Japan). For all analyses, significance was defined as *P* < 0.05.

## Results

### Patient characteristics and overall outcomes

**Figure 1:**
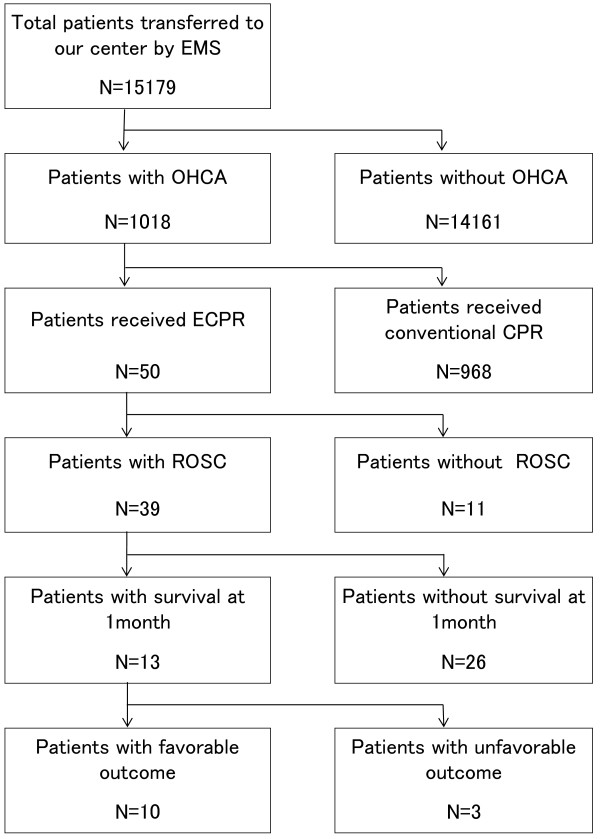
**Study flow chart and outcomes.** *EMS* emergency medical service, *ECPR* extracorporeal cardiopulmonary resuscitation, *CPR* cardiopulmonary resuscitation, *ROSC* return of spontaneous circulation.

During the study period, a total of 1,018 OHCA patients were transported to our center by ambulance. Of those, 50 underwent ECPR (Figure 1). Eight (three hypothermic and five young patients) fulfilled the criteria for the initiation of ECPR. However, these eight patients did not fulfill the exclusion criteria for the initiation of ECPR (one patient with severe trauma, four with irreversible brain damage, and three with activity-of-daily-living disability), and all of them had unfavorable outcomes.

Table 1 shows the characteristics of the 50 patients. Of these, 25 patients fulfilled the criteria for the initiation of ECPR, whereas ECPR was initiated in the other 25 patients according to the attending physician's judgment. Forty-seven (94%) were transported directly from the scene to our center, and three had inter-hospital transport. Nine patients (18%) were aged >70 years. Fourteen CA events were unwitnessed, and 18 patients did not receive bystander CPR. The majority of presumed causes of OHCA were cardiac etiologies: 17 patients (34%) with acute coronary syndrome, 8 (16%) with myocardial diseases, and 7 (14%) with arrhythmia. Seven patients (14%) experienced accidental hypothermia, three (6%) near-drowning, and two (4%) asphyxia by avalanche. The remaining six patients (12%) had other etiologies: respiratory arrest due to cerebellar hemorrhage (one patient), pulmonary embolism (two patients), acute aortic dissection (one patient), pesticide poisoning (one patient), and unknown cause (one patient). The majority of initial cardiac rhythms on the scene and on arrival at the center were VF.

**Table 1:** **Patient characteristics**

	Total (***n*** = 50)
Age, (years)	51 ± 21
Range	10–83
Male, *n* (%)	33 (66)
Witnessed, *n* (%)	36 (72)
Bystander CPR, *n* (%)	32 (64)
Initial rhythms at the scene	
VF/pulseless VT, *n* (%)	37 (74)
Asystole/PEA, *n* (%)	13 (26)
Initial rhythms on arrival	
VF/pulseless VT, *n* (%)	27 (54)
Asystole/PEA, *n* (%)	23 (46)
BLS-TOR rule	
Satisfied, *n* (%)	7 (14)
Unsatisfied, *n* (%)	43 (86)
ALS-TOR rule	
Satisfied, *n* (%)	1 (2)
Unsatisfied, *n* (%)	49 (96)
Comorbid diseases	
Cardiac diseases, *n* (%)	13 (26)
Other, *n* (%)	3 (6)
Circulatory arrest time, min	7.4 ± 10.4
CPR duration, min	84 ± 48
Door to ECPR initiation, min	37 ± 22
ECMO duration, *h*	38 ± 49

Data are presented as *n* (%) or mean ± standard deviation. *CPR* cardiopulmonary resuscitation, *VF* ventricular fibrillation, *VT* ventricular tachycardia *PEA* pulseless electrical activity, *BLS* basic life support, *ALS* advanced life support, *TOR* termination of resuscitation, *ECPR* extracorporeal cardiopulmonary resuscitation, *ECMO* extracorporeal membrane oxygenation.

Of the 50 patients, 13 (26%) had a history of cardiac diseases, and 3 (6%) had other comorbid diseases including transient ischemic attack (1 patient), aortic abdominal aneurysm (1 patient), and sleep apnea syndrome (1 patient).

One patient (12 years of age) required femoral cut-down procedures for cannulation. Six patients (12%) had ECPR-related complications. Three (6%) could not continue ECPR after 2 to 6 h due to hypovolemia following retroperitoneal hemorrhage. Three patients (6%) had lower limb ischemia requiring anterograde reperfusion. No patients had massive hemorrhage or obvious infections at the cannula insertion sites. Of the 50 patients who underwent ECPR, 16 received therapeutic hypothermia, 12 underwent PCI, and 21 underwent treatment with IABP.

**Figure 2:**
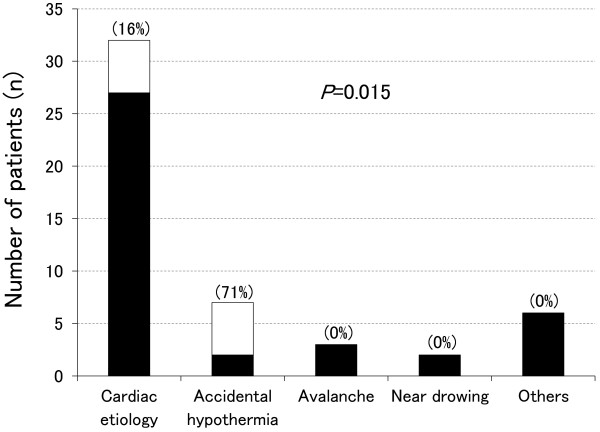
**Cause-specific outcomes of the 50 OHCA patients in whom ECPR was implemented.** The *open squares* indicate favorable outcomes, and the *blackened squares* indicate unfavorable outcomes. The *number in parentheses* indicates the percentage of favorable outcomes. *P* value indicates comparison of causes of cardiac arrest between the favorable outcome group and the unfavorable outcome group.

Among these 50 OHCA patients, 39 (78%) had ROSC. Thirteen (26%) survived 1 month after CA. Ten (20%) had favorable outcomes, with CPC 1. The remaining three had unfavorable outcomes, with CPC 3 (one patient) or CPC 4 (two patients). Of the 14 unwitnessed patients, 11 had unfavorable outcomes and 3 had favorable outcomes. All three of these patients with favorable outcomes experienced OHCA of hypothermic origin.Of the 32 patients with OHCA of cardiac origin, 5 were unwitnessed CA, and 11 did not have bystander CPR. Twenty-four patients (75%) had ROSC. Eight patients (25%) survived 1 month after CA. Five patients (16%) had favorable outcomes, with CPC 1 (Figure 2). The remaining three survivors had unfavorable outcomes, with CPC 3 (one patient) or CPC 4 (two patients). All five unwitnessed OHCA patients had unfavorable outcomes.

Of the seven patients with OHCA of hypothermic origin, all (100%) had ROSC. Five of the seven had unwitnessed CA, and four did not have bystander CPR. Five (71%) survived 1 month after CA, and all five survivors had favorable outcomes, with CPC 1.

By contrast, all patients with CA due to asphyxia by avalanche (two patients), near-drowning (three patients), and other causes (six patients) had unfavorable outcomes (Figure 2, *P* = 0.015).

### Comparison of patient characteristics and pre- and in-hospital variables

Table 2 shows the pre-hospital and in-hospital variables of the favorable outcome group compared with the unfavorable outcome group. Mean age and sex did not significantly differ between the groups. However, all nine patients over 70 years of age had unfavorable outcomes (*P* = 0.224).

**Table 2:** **Comparison between the favorable and unfavorable outcome groups**

	Favorable outcome (***n*** = 10)	Unfavorable outcome (***n*** = 40)	***P***value
Age, years	48 ± 18	52 ± 22	0.642
10–29 years, *n* (%)	2 (4)	7 (14)	0.224
30–49 years, *n* (%)	2 (4)	12 (24)	
50–69 years, *n* (%)	6 (12)	12 (24)	
70+ years, *n* (%)	0 (0)	9 (18)	
Male, *n*	5	28	0.277
Witnessed, *n* (%)	7 (14)	29 (58)	1.000
Bystander CPR, *n* (%)	4 (8)	28 (56)	0.138
Initial rhythms at the scene			
VF/pulseless VT, *n* (%)	9 (18)	28 (56)	0.258
Asystole/PEA, *n* (%)	1 (2)	12 (24)	
Initial rhythms on arrival			
VF/pulseless VT, *n* (%)	8 (16)	19 (38)	0.085
Asystole/PEA, *n* (%)	2 (4)	21 (42)	
BLS-TOR rule			
Satisfied, *n* (%)	0 (0)	7 (14)	0.319
Unsatisfied, *n* (%)	10 (20)	33 (66)	
ALS-TOR rule			
Satisfied, *n* (%)	0 (0)	1 (2)	1.000
Unsatisfied, *n* (%)	10 (20)	39 (78)	
Comorbid diseases			
Cardiac diseases, *n* (%)	1 (2)	12 (24)	0.258
Other, *n* (%)	1 (2)	2 (4)	0.496
Complications of ECPR, *n* (%)	0 (0)	6 (12)	0.571
Body temperature, °C	29.9 ± 6.1	31.8 ± 4.7	0.303
Pupil diameter, mm	5.1 ± 2.0	5.4 ± 1.4	0.501
Positive light reflex, *n* (%)	2 (4)	6 (12)	0.653
Laboratory data on arrival			
Arterial blood pH	7.10 ± 0.08	6.87 ± 0.25	<0.001
PaCO_2_, mmHg	52 ± 21	85 ± 37	0.010
PaO_2_, mmHg	150 ± 129	86 ± 99	0.096
Base excess, mEq/L	−14.7 ± 3.8	−19.3 ± 7.7	0.014
Lactate, mg/dL	101 ± 27	136 ± 56	0.061
Serum potassium, mEq/L	4.7 ± 1.7	4.8 ± 1.6	0.768
Circulatory arrest time, min	4.7 ± 5.8	8.1 ± 11.2	0.355
CPR duration, min	78 ± 46	85 ± 49	0.647
Door to ECPR initiation, min	30 ± 20	39 ± 23	0.231
ECMO duration, h	46 ± 46	36 ± 49	0.536

Data are presented as *n* (%) or mean ± standard deviation. *CPR* cardiopulmonary resuscitation, *VF* ventricular fibrillation, *VT* ventricular tachycardia, *PEA* pulseless electrical activity, *BLS* basic life support, *ALS* advanced life support, *TOR* termination of resuscitation, *ECPR* extracorporeal cardio-pulmonary resuscitation, *ECMO* extracorporeal membrane oxygenation.

No significant differences were observed in the rates of witnessed CA and bystander CPR between the groups. Initial rhythms at the scene and on arrival at the center were not significantly different between the groups. However, 12 (92%) of the 13 patients with unshockable rhythm at the scene had unfavorable outcomes. Only one OHCA patient of hypothermic origin with unshockable rhythm had a favorable outcome. In addition, all seven patients who satisfied the BLS-TOR rule had unfavorable outcomes. One patient who satisfied the ALS-TOR rule had also an unfavorable outcome. The groups did not significantly differ in body temperature on arrival at the center, pupil diameter, or the rate of positive light reflex.

With respect to laboratory findings on arrival, the favorable outcome group had significantly higher arterial pH (temperature-corrected values, *P* < 0.001), lower PaCO_2_ (*P* = 0.010), and higher base excess (*P* = 0.014) than the unfavorable outcome group. However, binary logistic regression analysis to compare the significant values with the presumed causes of OHCA revealed that there was no significant independent factor associated with outcomes (data are not shown in the tables). No significant differences in PaO_2_, serum lactate, or potassium values were observed between the groups.

Presumed circulatory arrest time (*P* = 0.355), CPR duration (*P* = 0.647), door to ECPR initiation time (*P* = 0.231), and ECMO duration (*P* = 0.536) did not significantly differ between the groups.

## Discussion

The use of ECPR for OHCA patients of heterogeneous origin in the only tertiary care center in a rural region rescued 26% of the patients who most likely would have died, and 20% had neurologically favorable outcomes. Of the patients with OHCA of cardiac origin, 16% had neurologically favorable outcomes. Of those with OHCA of non-asphyxiated hypothermic origin, 71% had neurologically favorable outcomes.

### Comparison of the effects on neurological outcomes between the present study and previous reports

The survival of OHCA patients differs between urban and rural areas [14–17]. Vukmir et al. found that the survival was highest in urban sites compared to that in suburban and rural sites [14]. According to a report by the Victorian Ambulance Cardiac Arrest Registry in 2012, an OHCA patient in a metropolitan region had at least a 1.5-times greater chance of survival at hospital discharge compared to an OHCA patient in a rural region [16]. In addition, Yasunaga et al. found that living in a sparsely populated area was associated with a low OHCA survival rate using the All-Japan Utstein-style registry database [17]. The difference in survival among OHCA patients between rural and urban areas is presumably due to the disparities in medical resources, which may affect response times, EMS resources, and the presence of hospitals with PCI capability. Our study was performed in a rural region consisting of small cities and vast rural and intermountain areas. Thus, the mean CPR duration in the present study was long (84 min). In addition, of all the patients with OHCA of cardiac origin in the present study, 16% experienced unwitnessed CA. A crucial predictor of OHCA outcome is whether the CA is witnessed or not [18]. Although we included the unwitnessed OHCA patients in the present study, the favorable outcome rate of patients with OHCA of cardiac origin was comparable to those reported by Nagao et al. [6] and Maekawa et al. [7], who investigated patients with witnessed OHCA in metropolitan areas. Our findings suggest that even in a sparsely populated area, ECPR is effective for rescuing patients with OHCA of cardiac origin.

A prolonged CPR duration is justified in such cases, and full neurological recovery has been described when ECPR is used in patients with OHCA of non-asphyxiated hypothermic origin [19, 20]. Farstad et al. reported that of 11 patients with OHCA of non-asphyxiated hypothermic origin in whom ECPR was implemented, 7 (64%) were neurologically intact survivors. In contrast, of 15 hypothermic OHCA patients with asphyxia due to avalanche or near-drowning, only 1 (7%) survived with a severe neurological deficit [20]. Our outcomes in OHCA patients with non-asphyxiated hypothermia are consistent with the high survival rate reported by Farstad et al. [20]. In addition, the outcomes of OHCA patients with asphyxia due to avalanche or near-drowning in this study are consistent with those of Farstad et al. [20].

Our findings suggest that the implementation of ECPR for patients with OHCA of cardiac origin and hypothermic origin in a rural region—where the CA bypass campaign has been gradually implemented—may lead to favorable neurological outcomes, similar to that observed in urban regions.

### Need for a regionalized care system for OHCA

The National Association of EMS Physicians in the USA has proposed that resuscitation efforts can be terminated in patients who do not respond after at least 20 min of CPR [21, 22]. All the patients with OHCA of cardiac origin in this study received CPR for longer than 33 min. These findings suggest that resuscitation efforts should not be terminated after the continuation of CPR for at least 20 min if ECPR is available. In the present study, the longest CPR duration in an adult patient with OHCA of cardiac origin who achieved a favorable outcome was 46 min. In addition, a 12-year-old child with hypertrophic cardiomyopathy receiving 81-min CPR during the winter after sudden CA had a favorable outcome. In contrast, the longest CPR duration in a patient with OHCA of hypothermic origin who achieved a favorable outcome was 165 min. The findings of this study and a previous report [20] suggest that even if the CPR duration exceeds 60 min in cases of OHCA in young patients or cases of non-asphyxiated hypothermic origin, there is a likelihood of survival without neurological damage if ECPR is implemented.

Of the 50 OHCA patients in whom ECPR was implemented in the present study, 94% were transported directly from the scene to our center. Before the CA bypass campaign was introduced in this region, EMS personnel typically transported OHCA patients from the scene of the CA to the nearest non-tertiary hospital. At that hospital, a physician unskilled in ALS usually attempted to resuscitate the OHCA patient. This situation remains unchanged in regions that still have a marked shortage of physicians. In contrast, advanced medical interventions such as defibrillation, tracheal intubation, and intravenous epinephrine administration have been made available as lifesaving techniques to EMS personnel at the scene of the OHCA, over the past 10 years. Thus, the lifesaving techniques are not greatly advanced in a non-tertiary hospital compared to that available to EMS personnel. However, large inter-hospital disparities have been reported in OHCA survival [23–25]. Wnent et al. found that OHCA patients who were transported to hospitals with PCI capability had better outcomes compared with those transported to non-PCI hospitals [24]. Similarly, Stub et al. demonstrated that a greater proportion of patients were discharged to their home after OHCA when admitted to a facility with PCI capability [25]. These findings suggest that the characteristics of the receiving hospital determine the patient outcomes following OHCA. Carr et al. reported that large and teaching hospitals demonstrated lower post-CA mortality compared to small and non-teaching hospitals [26]. Therefore, as the only university tertiary care center in our rural region, we have been promoting the CA bypass campaign, which is consistent with the proposal by the American Heart Association (AHA) for the implementation of regionalized care systems for OHCA [27].

However, there is no doubt that a shorter CPR duration is associated with a better survival rate in ECPR [28–30]. Haneya et al. demonstrated that a longer CPR duration was associated with poor outcome [28]. The survival rate was higher for cases of CPR with duration of less than 30 min than for CPR with duration of more than 30 min [28]. CPR duration of more than 60 min rarely results in favorable outcomes in patients with OHCA of cardiac origin [30].

Although the effects of the CA bypass campaign remain unclear, regionalized care systems for the immediate initiation of ECPR in OHCA patients who are unresponsive to conventional CPR are considered to be necessary in regions with limited medical resources.

### Predictors of neurologically favorable outcomes

Nonetheless, no clinically reliable predictors are available to identify ECPR candidates. Müller et al. described that only witnessed OHCA with immediate-bystander CPR should be considered eligible for ECPR [31]. However, this is not the case for OHCA patients of non-asphyxiated hypothermic origin, as suggested in this study. In this study, three of the five OHCA patients of hypothermic origin with favorable outcomes had unwitnessed CA. When discussing ECPR candidates, the etiologies of OHCA should be clarified. Neurological outcomes differ according to the etiology of CA [20, 28].

No differences in patient characteristics or pre- and in-hospital variables were observed in this study, with the exception of pH, PaCO_2_, and base excess. These findings are consistent with those obtained after establishing ECPR in children [5] and adults [28]. However, these factors were not significantly and independently associated with outcomes in the present study, and the values of pH, PaCO_2_, and base excess on arrival are not helpful for identifying an ECPR candidate. The same is true of pupil diameter on arrival, which is suggested to be a key predictor to identify ECPR candidates [7]. We did not find any difference in pupil diameter in this study.

Advanced age is often considered a contraindication for ECPR support, although this recommendation is controversial. Probably due to the relatively small sample size, we did not see an increased unfavorable outcome rate in patients of older age. These results are consistent with a previous study [28]. However, all of the OHCA patients over 70 years old in whom ECPR was implemented in this study had unfavorable outcomes.

In Japan, EMS personnel are legally prohibited from terminating CPR in the field. Thus, the BLS- and ALS-TOR rules are not common. In this study, all seven patients who satisfied the BLS-TOR rule at the scene had unfavorable outcomes. One patient who satisfied the ALS-TOR rule also had an unfavorable outcome. The BLS- and ALS-TOR rules may be used as exclusion criteria for ECPR.

### Limitations

This study has several limitations. First, this study is a single-center experience in a single region. The generalizability of these findings is limited. Second, the number of patients we studied is relatively small. In addition, the retrospective observational design and the heterogeneity of our study population precluded our ability to detect predictors that may have influenced our outcomes. Third, this region is located near the Japanese Alps, where the monthly mean lowest temperatures drop to −2°C to −5°C during the 3 months of winter. Sugita et al. reported that in children, neurological outcomes after CA were better in winter than in summer [32]. In addition, intra-arrest hypothermia is suggested to improve neurological outcome [33]. Thus, these findings may not be similarly applicable in hot regions throughout the year. Fourth, we could not conduct a comparative study by using data before and after the implementation of the CA bypass campaign. Our center was established approximately 10 years ago, at approximately the same time as the concept of CA bypass was initiated in this region. Before the initiation of this campaign, OHCA patients were rarely transported to our university hospital. If this campaign was not implemented, the OHCA patients who received ECPR in the present study would have been most likely transported from the scene of the CA to the nearest hospital. Thus, the actual effects of the CA bypass campaign on OHCA patients remain to be elucidated.

## Conclusions

The 20% overall rate of favorable neurological outcomes in a rural tertiary care center demonstrates that ECPR is a useful modality to rescue patients with OHCA of heterogeneous origin that does not respond to conventional CPR, similar to that noted in urban regions. Further investigations of regionalized care systems for OHCA are deemed necessary in regions with limited medical resources.

## Authors' information

KM is a graduate student of the Department of Emergency and Critical Care Medicine, Shinshu University School of Medicine, Japan. HI and TI are Associate Professors at the Department of Emergency and Critical Care Medicine, Shinshu University School of Medicine, Japan. KO is a Professor and Chairman of the Department of Emergency and Critical Care Medicine, Shinshu University School of Medicine, Japan.
